# Family support, anger and aggression in health workers during the first wave of the pandemic

**DOI:** 10.3934/publichealth.2023037

**Published:** 2023-06-15

**Authors:** Argyro Pachi, Maria Anagnostopoulou, Athanasios Antoniou, Styliani Maria Papageorgiou, Effrosyni Tsomaka, Christos Sikaras, Ioannis Ilias, Athanasios Tselebis

**Affiliations:** 1 Psychiatric Department, Sotiria Thoracic Diseases Hospital of Athens, 11527 Athens, Greece; 2 Nursing Department, Sotiria Thoracic Diseases Hospital of Athens, 11527 Athens, Greece; 3 Department of Endocrinology, “Elena Venizelou” Hospital, 11521 Athens, Greece

**Keywords:** anger, aggression, family support, healthcare workers, physicians, nurses, COVID-19 pandemic

## Abstract

**Introduction:**

Anger is considered as one of the basic human emotions, constituting the affective component of aggression. In the first year of the pandemic, the intense pressure on healthcare workers resulted in the deterioration of their psychosocial problems.

**Objective:**

The aim of this study is to investigate the relationship between family support, anger, and aggression.

**Methods:**

The present study included physicians and nurses who completed an online survey of Dimensions of Anger Reactions-5 (DAR-5), a Brief Aggression Questionnaire (BAQ) and a Family Support Scale (FSS). Before completing the questionnaires, participants were asked to state their gender, years of work, age, and profession.

**Results:**

Fifty-three men and 190 women participated in the study. Almost one-third of the participants had a positive score on the DAR-5 scale. Male participants displayed lower DAR-5 scores compared to women. Female participants displayed lower FSS scores compared to men, but higher scores when compared with earlier measures. Regression showed that 15.2% of the variance in BAQ scores can be explained by DAR-5 scores, with an additional 3.8% explained by FSS scores, while an additional 2.3% is explained by years of working experience. Mediation analysis highlighted the role of family support as a negative mediator in the DAR-5 and BAQ relationship.

**Conclusion:**

During the first year of the pandemic, there was an increase in the sense of family support among female health workers. One-third of the participants displayed increased anger scores. Family support acts as a mediator by preventing anger derailing into aggression. In healthcare worker support programs, it seems necessary to entail a specific section on anger management.

## 1. Introduction

The first wave of the COVID-19 pandemic in 2020 had profound implications for both the general population and healthcare workers worldwide [1]–[3]. The difficult working conditions of healthcare workers including shortages of personal protective equipment, fear of infecting their loved ones [4], and the simultaneous pressure of severe containment measures resulted in the deterioration of their psychosocial problems [4].

Anger is considered as one of the basic emotions of [5] humans, mainly due to its distinct and universally recognizable pattern of facial expression, posture, and gestures [6]. Expression of anger on the face can be interpreted as a sign of aggression, causing either fear reactions or a tendency to engage in a conflict. Many researchers argue that the expression of anger occurs in infants, even by the end of the first year of life [7], while they believe that anger is necessary to protect the individual from potential dangers [7]. This basic protective role of the anger response gradually evolve into a more complex response sequelae that is activated by the perception of a threat in the external world and is useful for initiating and supporting the fight-flight response [6].

In summary, anger is a basic human emotion that involves a complex set of physiological and psychological responses to perceived threats, frustrations, or injustices [8]. It is characterized by feelings of resentment, hostility, and arousal, as well as cognitive appraisals that trigger an impulse for an aggressive or assertive response [9],[10]. Anger can vary in intensity, from mild irritation to explosive rage, and can have positive and negative effects on individuals and their relationships with others [11]. Aggression can be defined as “any behavior that is intended to cause harm to another person” [12]–[14]. This can manifest itself in a variety of forms, such as physical violence, verbal attacks or other hostile behaviors.

Anger and aggression are two closely related concepts, but they are not synonymous. Anger refers to an emotional state characterized by feelings of annoyance, frustration, or dissatisfaction, while aggression refers to behavior intended to cause harm. The emotional experience of anger does not always lead to an aggressive behavior [15].

A widely accepted model of aggression is the General Aggression Model (GAM), which proposes that aggression is the result of a combination of biological situational and individual factors, including personal characteristics, past experiences, and environmental cues [16].

By family support, we mean the sense of support a person has from the other people with whom he or she lives [17]. The role of family support became more important during the pandemic because of the stringent quarantine restrictions, since the role of other types of social support was reduced during quarantine [17]. Studies have shown a beneficial effect of family support in patients with chronic diseases such as chronic obstructive pulmonary disease [18], bronchial asthma [19], and diabetes mellitus [20], as well as in patients with lung cancer [21].

What is missing from the relevant studies is a definition of the role of family support as a factor that may counteract anger, influencing its experience and expression and prevent its derailment into aggression. The purpose of this study is to investigate the relationship between family support, anger, and aggression.

## 2. Materials and methods

This is a cross-correlational study. Self-report questionnaires were used for data collection. These questionnaires were sent via email. The email invitation contained an anonymous link that allowed access to the online survey platform. The email addresses of the participants were retrieved through links to websites of Greek healthcare workers from their scientific and professional societies. On the first page of the electronic questionnaire, we assured that a) participation in the survey was voluntary and b) completion and submission of the questionnaire was considered to be a declaration of consent. The study sample included medical and nursing staff who agreed to respond to the email. The study was conducted from June 15 to June 30, 2020. Until the end of the study, the cumulative confirmed COVID-19 deaths did not exceed 200, while the diagnosed cases did not exceed 3500. The low infection rate in the population during the first wave was likely due to the lockdown that was imposed throughout Greece, starting on March 23. The specific lockdown was characterized as one of the strictest in Europe. From May 4, a plan for the gradual de-escalation of the restrictive measures was implemented.

### 2.1. Study participants

An invitation to participate in the study was sent to 150 doctors and 250 nurses, 243 of whom (120 doctors and 123 nurses) responded to the invitation. Sample adequacy was calculated using the G-Power software [22],[23]. With a sample of 243 subjects, seven factors, and an alpha of 0.05, the calculated power was 1.00. A Monte Carlo power analysis was performed for a single-mediated model [24],[25]. For a sample of 243 subjects, 5000 replications, and a 95% Confidence Level, the calculated power was 0.9. In the present study, no measures were taken to increase the response rate, apart from a reassurance of data privacy.

### 2.2. Measurement tools

Before completing the questionnaires, participants were asked to indicate their occupation, gender, years of work, and age. Then, the healthcare workers completed the following questionnaires, listed below.

#### 2.2.1. Dimensions of Anger Reactions-5 (DAR-5)

The DAR-5 Anger Scale is a short, 5-item scale that measures the anger experience during the past 4 weeks. Respondents rate their anger experience on a 5-point Likert scale ranging from 1 = never or almost never to 5 = always or almost always. The five scores are summed, with a total score ranging from 5 to 25. Higher scores indicate a more severe anger experience. The cut-off point for the scale is ≥12 [26],[27]. Regarding the questionnaire's internal reliability in the present study, Cronbach's alpha was equal to 0.759.

#### 2.2.2. Brief Aggression Questionnaire (BAQ)

The Brief Aggression Questionnaire (BAQ) is a 12-item self-report psychometric instrument on aggression. The questionnaire asks participants to rate, the extent to which the statements are typical of themselves on a scale of 1 (strongly agree) to 5 (strongly disagree). The BAQ has been proposed as a valid and reliable instrument, with a good retest reliability and convergent validity compared to other aggression instruments [28]–[30]. The internal reliability in the present study had a Cronbach's alpha of 0.762.

#### 2.2.3. Family Support Scale (FSS)

The Family Support Scale (FSS) aims to capture the sense of support a subject receives from his/her family members (with whom he/she lives). The scale consists of 13 items, which are answered on a Likert scale, ranging from 1 (“strongly disagree”) to 5 (“strongly agree”). The scale is self-administered, and all items focus on the relationships between people living together [17],[19],[31]. High scores correspond to an increased sense of family support. People living alone did not complete the scale [31]. The internal reliability in the present study had a Cronbach's alpha of 0.786.

### 2.3. Ethical considerations

The study has been approved by the Clinical Research Ethics Committee of “Sotiria” General Hospital (Number 12253/7–5–20). This study was conducted in accordance with the ethical principles as defined by the Declaration of Helsinki, the International Committee of Medical Journal Editors, and the General Data Protection Regulation (GDPR–2016/679) of the European Union.

### 2.4. Statistical analysis

Data were first analyzed based on descriptive statistics, and continuous variables were expressed as mean and standard deviation. Then, data were analyzed based on inferential statistics. To test for differences between independent samples, such as gender, and differences between healthcare workers, the independent samples t-test was used. Pearson's correlation was used to determine the strength and direction of the associations between variables. Linear regression models were constructed to investigate whether the associated variables were significant predictors of aggression. Analyses were performed using IBM SPSS 20. Mediation analyses were performed using the Hayes SPSS Process Macro [32],[33]. The outcome variable for the analysis was BAQ. The predictor variable for the analysis was DAR-5. The mediation variable for the analysis was FSS. Statistical significance was set at p < 0.05.

## 3. Results

A total of 53 men and 190 women participated in the study (Table 1). A positive score value on the DAR-5 (DAR-5 ≥ 12) questionnaire was reported from 32.5% of the participants. Men scored significantly lower on the DAR-5 scale compared to women (9.38 ± 2.52 vs. 10.37 ± 3.50, t-test p < 0.05, Hedges' g: 0.30) (Table 1). Women scored significantly lower on the FSS compared to men (49.53 ± 8.62 vs. 52.02 ± 6.38, t-test p < 0.05, Hedges' g: 0.28) (Table 1).

**Table 1.** General characteristics of healthcare personnel and DAR-5, BAQ & FSS scores as to gender.

Gender		Age	Work experience (in years)	Dimensions of Anger Reactions (DAR-5)	Brief Aggression Questionnaire (BAQ)	Family Support Scale (FSS)
Male	Mean	42.11	13.23	9.38*	24.34	52.02*
	N	53	53	53	53	49
	Std. Deviation	9.89	11.05	2.52	5.57	6.38
Female	Mean	41.49	15.54	10.37*	23.33	49.53*
	N	190	190	190	190	182
	Std. Deviation	9.38	10.58	3.50	7.27	8.62
Total	Mean	41.63	15.03	10.16	23.55	50.06
	N	243	243	243	243	231
	Std. Deviation	9.48	10.71	3.33	6.93	8.25

*Note: *p < 0.05.

Women displayed higher mean FSS scores (49.53 ± 8.62 vs. 47.00 ± 9.8 unpaired t-test, two-tailed p < 0.05) compared with earlier measurements [31]. Nurses were older and had more years of professional experience compared to physicians (t-test p < 0.01, Table 2), but no significant differences on scores in the administered questionnaires were observed (t-test p > 0.05, Table 2).

**Table 2.** General characteristics of healthcare staff and DAR-5, BAQ & FSS scores as to profession.

Profession		Age	Work experience (in years)	Dimensions of Anger Reactions (DAR-5)	Brief Aggression Questionnaire (BAQ)	Family Support Scale (FSS)
Physicians	Mean	38.39*	9.84*	10.00	24.12	50.66
	N	120	119	120	120	112
	Std. Deviation	9.48	9.02	3.12	7.04	7.76
Nurses	Mean	44.79*	20.09*	10.31	22.98	49.49
	N	123	123	123	123	119
	Std. Deviation	8.38	9.78	3.53	6.80	8.67

*Note: *p < 0.01.

Compared to the rest of the sample, participants living alone did not present a statistical difference (t-test p > 0.05) on scores both in the BAQ scale (23.58 ± 7.62 vs. 23.55 ± 6.91) and in the DAR-5 scale (9.83 ± 2.79 vs. 10.17 ± 3.36).

High negative correlations were observed between FSS with both DAR-5 and BAQ (Table 3). High positive correlations were evidenced between DAR-5 and BAQ (Table 3). Age and work experience showed negative relationships with DAR-5 (Table 3).

**Table 3.** Correlations among age, work experience (in years), DAR-5, BAQ and FSS.

Pearson correlation		Age	Work experience (in years)	Dimensions of Anger Reactions (DAR-5)	Brief Aggression Questionnaire (BAQ)
Work experience (in years)	r	0.861**			
	Sig. (2-tailed)	0.001			
	N	241			
Dimensions of Anger Reactions (DAR-5)	r	−0.002	0.058		
	Sig. (2-tailed)	0.981	0.367		
	N	243	243		
Brief Aggression Questionnaire (BAQ)	r	−0.144*	−0.144*	0.403**	
	Sig. (2-tailed)	0.025	0.025	0.001	
	N	243	243	243	
Family Support Scale (FSS)	r	−0.025	0.032	−0.245**	−0.280**
	Sig. (2-tailed)	0.701	0.631	0.001	0.001
	N	231	231	231	231

*Note: *Correlation is significant at the 0.05 level (2-tailed); **Correlation is significant at the 0.01 level (2-tailed).

We then performed a multiple regression analysis (Stepwise method) to identify the factors that best explained BAQ scores. Specifically, we set the BAQ as the dependent variable and as independent variables: gender, age, work experience, occupation, and DAR-5 and FSS scores. Visual inspection of the normal P-P plot of regression standardized residual with the dependent variable BAQ showed that the regression residuals followed a normal distribution. Homoscedasticity was examined by visual inspection of the scatter plot of regression standardized residuals and regression standardized predicted values. This regression analysis showed that 15.2% of the variance in the BAQ scores can be explained by the DAR-5 scores, an additional 3.8% is explained by the FSS scores, and an additional 2.3% is explained by work experience; the other variables did not explain the variance in BAQ (Table 4).

**Table 4.** Stepwise multiple regression analysis of factors predicting Brief Aggression Questionnaire (only statistically significant variables are included).

Dependent Variable: Brief Aggression Questionnaire	R Square	R Square Change	Beta	t	p	VIF	Durbin-Watson
Dimensions of Anger Reactions (DAR-5)	0.152	0.152	0.351	5.746	0.001*	1.066	2.278
Family Support Scale (FSS)	0.189	0.038	−0.193	−3.155	0.001*	1.064	
Work experience (in years)	0.213	0.023	−0.153	−2.585	0.01*	1.001	

*Note: Beta = standardized regression coefficient; correlations are statistically significant at the *p < 0.01 level.

**Figure 1.**
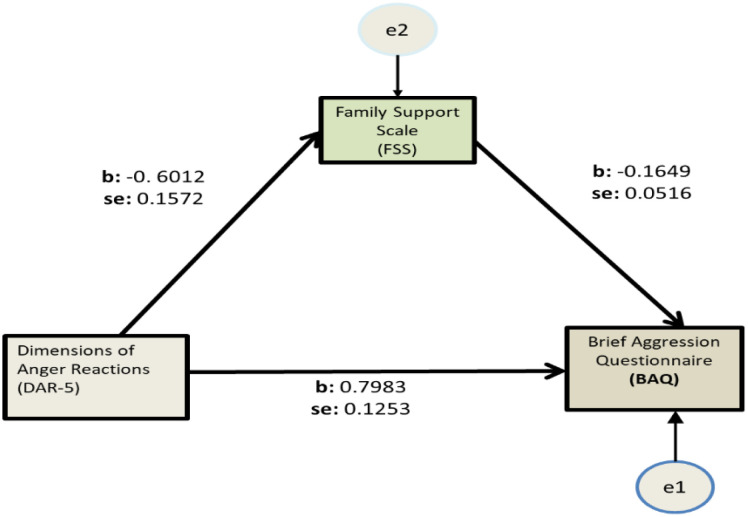
Mediation analysis of Family Support Scale (FSS) on Dimensions of Anger Reactions (DAR-5) - Brief Aggression Questionnaire (BAQ).

Bootstrapping was performed with the Hayes SPSS Process Macro (Model 4) to examine whether FSS mediated the relationship between DAR-5 and BAQ, based on 5000 bootstrap samples (Table 5, Figure 1).

**Table 5.** Mediation analysis of Family Support Scale (FSS) on Dimensions of Anger Reactions (DAR-5) - Brief Aggression Questionnaire (BAQ) relationship.

Variable	b	SE	t	p	95% Confidence Interval	
					LLCI	ULCI
DAR-5→FSS	−0. 6012	0.1572	−3.8238	0.001	−0. 9109	−0.2914
DAR-5→BAQ	0.7983	0.1253	6.3706	0.001	0.5514	1.0452
DAR-5→FSS → BAQ	−0.1649	0.0516	−3.1928	0.016	−0.2666	−0.0631
Effects
Direct	0.6992	0.1267	5.5170	0.001	0.4494	0.9489
Indirect**	0.0991	0.0410			0.0284	0.1878
Total	0.7983	0.1253	6.3706	0.001	0.5514	1.0452

*Note: *Based on 5000 bootstrap samples; **Gender, Work Experience and Age were included in the analysis as covariates variables. They are not shown in the table as they did not give significant statistical results (p > 0.05).

A statistically significant indirect relationship was found, with family support having a negative effect on anger and aggression [B = 0.0991, 95% CI (0.0284, 0.1878) p < 0.05]. Figure 1 depicts the coefficients of the variables. The model explains 12.41% of the variance in the outcome variable.

## 4. Discussion

A common stereotype considers anger as an emotion that mainly affects the male population [34],[35]. The activation of this stereotype makes us incorrectly attribute female anger to internal causes and characteristics of women; in contrast, male anger is attributed to external environmental causes [36]. The female population of our sample showed higher anger scores than male healthcare workers and this result should be evaluated, considering the circumstances and significant shortages at the beginning of the pandemic [4],[37], in relation to the stereotypical perception of a female health worker as a person dedicated to offering.

In this study, a significant proportion of health workers admitted to experiencing strong feelings of anger. This finding is consistent with the literature, which suggests that increased levels of anger are associated with an increased number of negative life events. Thus, stressful situations, such as the pandemic and the constraints that accompanied it, effectuated high levels of anger in the general population [38],[39] and in healthcare workers [40]–[42]. It should also be emphasized that anger can be a symptom of both anxiety and depressive disorders, which are two disorders frequently found in healthcare workers [43],[44]. Poor anger management by health professionals can result in errors in the patient care [6] and can have an impact on the quality of healthcare services. Therefore, it is important to identify factors that may counteract anger.

It is particularly common in studies for the female population to express a lower sense of family support compared to the male population; this finding probably reflects typical features of Greek society in relation to formal societal roles that both sexes are called upon to assume [18],[19],[31]. The observed increase in the sense of family support in the female population compared to earlier measurements is most likely related to a number of media and political publications that portrayed health workers as heroes [45],[46].

The relationship between anger and aggression has been documented in numerous studies in both psychological and biological contexts [47],[48], concluding that these are not two identical concepts, and that anger does not inevitably lead to aggression. In the present study, 15% of the variance in aggression was explained by anger, and it seems that family support mediates this by preventing anger from turning into aggression. It is possible that there are more psychological factors hindering this diversion, so a sense of coherence is suggested in studies to impede the expression of aggression, at least in patients with schizophrenia [30]. Additionally, cross-cultural factors are likely to be involved in the expression of anger [49],[50].

According to literature, even before the pandemic, healthcare workers lacked sufficient anger management skills [51] and experienced intense negative emotions, burnout, psychological distress, anxiety, and depression [43],[52],[53]; these factors can contribute to increased anger and aggression. For those living with family members, interactions within the family may be protective against the negative effects of stress. However, the effectiveness of family support depends on factors such as the context surrounding support transactions, as well as the recipient's satisfaction with the support.

During the initial phase of the pandemic and with strict containment measures, there were strong fears of an increase in domestic violence [54],[55], which are fears that have been verified in subsequent studies [56],[57]. Certainly, the present study cannot dismiss the validity of these findings in health workers, but it seems unlikely that there is a simultaneous increase in family support and domestic violence.

Finally, it is important to note that the anger expression is associated with biological health risks, particularly coronary heart disease [58],[59]. Thus, we believe that it is important for the health of healthcare workers to implement support programs to address anger management issues.

The study attempted to examine the role of the sense of family support in the relationship between anger and aggression. Apart from family support, it is almost certain that there are other protective factors that limit the diversion of anger to aggression, such as psychological resilience and sense of coherence; on the other hand, there are negative factors such as depression, anxiety, and burnout, that increase aggression. Future research should examine the impact of these factors on aggression in order to have more effective intervention programs. Particularly in relation to anxiety and stress, it would be useful to use specific scales such as stress and anxiety to viral epidemics scale-9 [60],[61].

However, this study is also subject to limitations. Gender disproportionality of participants may affect the generalizability of the results, while conducting the study with self-administered questionnaires imparts a more subjective dimension to the assessment of the variables. Moreover, we did not consider whether health workers were currently allocated on the front line of the pandemic. Lastly, to follow pandemic guidelines, data was collected through an online method. This meant that healthcare professionals without internet access could not participate. Therefore, the data collected do not represent the views of these individuals and further influence the generalizability of the study.

## 5. Conclusions

During the first year of the pandemic, there was an increase in the sense of family support among female health workers. One third of participants in this study displayed high anger scores and the female population displayed higher anger scores than the male population. Family support exhibited a negative correlation with both anger and aggression and acts as a mediator by suppressing the diversion of anger into aggression. It is considered necessary that healthcare workers' support programs also encompass anger management issues.
